# The plastid genome of a tropical tree *Alseodaphne petiolaris* (Lauraceae)

**DOI:** 10.1080/23802359.2019.1676178

**Published:** 2019-10-12

**Authors:** Rongjie Zhu, Yu Song, Guanfei Zhao, Jie Yang, Xilong Wang

**Affiliations:** a Institute of Vegetable Sciences, Tibet Academy of Agricultural and Animal Husbandry Sciences, Tibet, China;; b Center for Integrative Conservation, Xishuangbanna Tropical Botanical Garden, Chinese Academy of Sciences, Yunnan, China;; c Tibet Science and Technology Department, Tibet Plateau Institute of Biology, Tibet, China

**Keywords:** *Alseodaphne*, chloroplast genome, phylogenetic analysis

## Abstract

*Alseodaphne petiolaris* Hook.f. is a valuable timber tree of the genus *Alseodaphne* Nees in the family Lauraceae. To better determine its phylogenetic location with respect to the other *Alseodaphne* species, the complete plastid genome of *A. petiolaris* was sequenced. The whole plastome is 152,986 bp in length, consisting of a pair of inverted repeat (IR) regions of 20,108 bp, one large single copy (LSC) region of 93,863 bp, and one small single copy (SSC) region of 18,907 bp. The overall GC content of the whole plastome is 39.1%. Further, maximum-likelihood phylogenetic analyse was conducted using 15 complete plastomes of the Lauraceae, which support the close relationship between *A. petiolaris* and the species of *Machilus* and *Phoebe*.

*Alseodaphne petiolaris* Hook.f., a tree species distributed in Yunnan of SW China, India, and Myanmar, was assigned to the genus *Alseodaphne* Nees in the family Lauraceae (http://foc.iplant.cn/). Several species of *Alseodaphne* are known for their high wood quality, such as *A. andersonii* (King ex Hook.f.) Kosterm., *A. semecarpifolia* Nees*, and A. petiolaris.* For a better understanding of the relationships of *A. petiolaris* and other *Alseodaphne* species, it is necessary to reconstruct a phylogenetic tree based on high-throughput sequencing approaches.

Fresh leaves of *A. petiolaris* in Ximeng County (Yunnan, China; Long. 99.60928 E, Lat. 22.58699 N, 1579 m) were picked for DNA extraction (Doyle and Dickson 1987). The voucher was deposited at the Biodiversity Research Group of Xishuangbanna Tropical Botanical Garden (Accession Number: XTBG-BRG-SY33137). The whole plastid genome was sequenced following Zhang et al. (2016), and their 15 universal primer pairs were used to perform long-range PCR for next-generation sequencing. The contigs were aligned using the publicly available plastid genome of *Persea americana* (GenBank accession number KX437771) (Song et al. 2016) and annotated in Geneious 4.8.

The plastome of *A. petiolaris* (LAU00099), with a length of 152,986, was 103 bp, 74 bp and 55 bp smaller than that of *A. gracilis* (153,099 bp, MG407593), *A. huanglianshanensis* (153,070 bp, MG407594) and *A. semecarpifolia* (153,051 bp, MG407595) (Song et al. 2018). It was also 256 bp and 110 bp larger than that of *Machilus balansae* (152,721 bp, KT348517) (Song et al. 2015) and *Phoebe sheareri* (152,876 bp, KX437773) (Song et al. 2017). The length of the inverted repeats (IRs), large single-copy (LSC), and small single-copy (SSC) regions of *A. petiolaris* was 20,108 bp, 93,863 bp, and 20,108 bp, respectively. The overall G + C content was 39.1%.

Furthermore, based on 15 published plastid genome sequences, we reconstructed a phylogenetic tree (Figure 1) to confirm the evolutionary relationship between *A. petiolaris* and other related species with published plastomes in *Alseodaphne*, *Machilus*, *Persea*, and *Phoebe*, with *Endiandra* species as outgroup. Maximum-likelihood (ML) phylogenetic analyses were performed base on TVM + F+R3 model in the iqtree version 1.6.7 programme with 1000 bootstrap replicates (Nguyen et al. 2015). The ML phylogenetic tree with 95% to 100% bootstrap values at each node supported that *Alseodaphne* species grouped into two clades and that *A. petiolaris* and the species of *Machilus* and *Phoebe* were located in the same clade, suggesting that *Alseodaphne* is polyphyletic.

**Figure 1.**
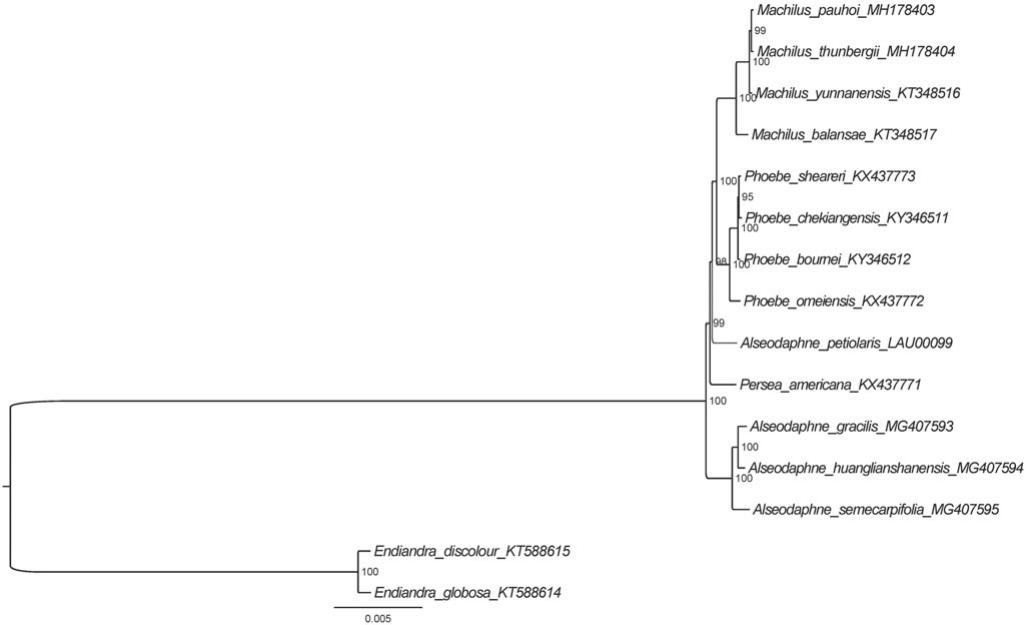
The ML phylogenetic tree for *A. petiolaris* based on other 15 species (four in *Alseodaphne*, two in *Endiandra*, four in *Machilus*, one in *Persea*, and four in *Phoebe*) plastid genomes.

## Data Availability

The plastome data of the *A. petiolaris* will be submitted to Lauraceae Chloroplast Genome Database (https://lcgdb.wordpress.com). Accession numbers are LAU00099.

## References

[CIT0001] Doyle JJ, Dickson EE 1987 Preservation of plant samples for DNA restriction endonuclease analysis. Taxon. 36(4):715–722.

[CIT0002] Nguyen LT, Schmidt HA, von Haeseler A, Minh BQ 2015 IQ-TREE: a fast and effective stochastic algorithm for estimating maximum likelihood phylogenies. Mol Bio Evol. 32(1):268–274. 25371430 10.1093/molbev/msu300 PMC4271533

[CIT0003] Song Y, Dong WP, Liu B, Xu C, Yao X, Gao J, Corlett RT 2015 Comparative analysis of complete chloroplast genome sequences of two tropical trees *Machilus yunnanensis* and *Machilus balansae* in the family Lauraceae. Front Plant Sci. 6:662. 26379689 10.3389/fpls.2015.00662 PMC4548089

[CIT0004] Song Y, Yao X, Liu B, Tan YH, Corlett RT 2018 Complete plastid genome sequences of three tropical *Alseodaphne* trees in the family Lauraceae. Holzforschung. 72(4):337–345.

[CIT0005] Song Y, Yao X, Tan YH, Gan Y, Corlett RT 2016 Complete chloroplast genome sequence of the avocado: gene organization, comparative analysis, and phylogenetic relationships with other Lauraceae. Can J for Res. 46(11):1293–1301.

[CIT0006] Song Y, Yao X, Tan YH, Gan Y, Yang JB, Corlett RT 2017 Comparative analysis of complete chloroplast genome sequences of two subtropical trees, *Phoebe sheareri* and *Phoebe omeiensis* (Lauraceae). Tree Genet Genomes. 13:120.

[CIT0007] Zhang T, Zeng CX, Yang JB, Li HT, Li DZ 2016 Fifteen novel universal primer pairs for sequencing whole chloroplast genomes and a primer pair for nuclear ribosomal DNAs. J Sytematics Evolution. 54(3):219–227.

